# The coadministration of *Lactobacillus* probiotic augments the antitumor effect of telmisartan in rats

**DOI:** 10.1186/s13568-025-01843-3

**Published:** 2025-03-05

**Authors:** Ahmed M. El-Baz, Amany A. El-Mahmoudy, Sameh Saber, Marwa T. ElRakaiby

**Affiliations:** 1 Department of Microbiology and Immunology, Faculty of Pharmacy, Delta University for Science and Technology, Gamasa, 11152 Egypt; 2 Dakahliya Health Directorate, Ministry of Health and Population, Dakahliya, 35931 Egypt; 3 Department of Pharmacology, Faculty of Pharmacy, Delta University for Science and Technology, Gamasa, 11152 Egypt; 4 Department of Microbiology and Immunology, Faculty of Pharmacy, Cairo University, Cairo, 11562 Egypt

**Keywords:** Gut microbiota, *Lactobacillus*, Colorectal cancer, VEGF, Annexin V, CEA

## Abstract

Colorectal cancer (CRC) is a prevalent disease with a high mortality rate and is significantly affected by microbial dysbiosis. Recent research suggests that modulation of the gut microbiome can have therapeutic benefits and that Angiotensin-II Type 1 Receptor (AT1R) can stimulate cell growth, angiogenesis, and resistance to apoptosis in various cancers. In this study, the adjunctive administration of *Lactobacillus* spp. and telmisartan, an AT1R blocker, was explored in the treatment of CRC. The effect of telmisartan and a mixture of probiotic species, *Lactobacillus delbrueckii* and *Lactobacillus fermentum*, was assessed on key biomarkers and selected gut microbiota taxa in 1,2-dimethylhydrazine-induced CRC in rats. Angiogenesis, inflammation, and apoptosis were assessed by measuring vascular endothelial growth factor (VEGF), carcinoembryonic antigen (CEA), Interleukin 6 (IL-6), and Annexin V levels, respectively. The relative abundance of selected gut microbial taxa, including *Bacteroides* spp.,* Clostridium* spp.,* Clostridium coccoides*,* Ruminococcus* spp., and *Lactobacillus* spp. was analyzed to determine the change in the microbial composition in the different experimental groups of the animal model. This study demonstrated that the unique combination therapy using a *Lactobacillus* mixture and telmisartan effectively reduced VEGF and IL-6 levels, indicating decreased angiogenesis and inflammation. *Lactobacillus* spp. co-administration with telmisartan boosted programmed cell death, reversed dysbiosis, improved histopathological outcomes, and reduced CEA levels. These findings offer a new perspective on the role of *Lactobacillus* spp. and telmisartan in CRC treatment. Further research on their adjunctive use and therapeutic potential are needed to enhance clinical efficacy.

## Introduction

Colorectal cancer (CRC) is the second most common cause of mortality worldwide, and it ranks third in terms of global cancer prevalence (Sung et al. 2021). The incidence of CRC is affected by hereditary and environmental factors (Mármol et al. 2017). The risk of developing CRC can be exacerbated by multiple predisposing factors, which include age, gender, type 2 diabetes, smoking, alcohol addiction, obesity, and a family history of CRC or adenomatous polyps (Demb et al. 2019; Mathers 2019). In addition, high blood pressure has a significant impact on the likelihood of developing colon cancer, particularly in men (Xuan et al. 2021).

The effect of the gut microbiome on the progression of CRC is increasingly gaining attention (Li et al. 2022). The maintenance of a balanced gut microbiota is essential for overall well-being while the disruption of the gut microbial composition and diversity can lead to dysbiosis and various diseases (Khalaf et al. 2022). There is a direct link between gut dysbiosis and the development of CRC (Artemev et al. 2022). In CRC treatment, the metabolic activity of the gut microbiome can alter the pharmacokinetics of chemotherapeutic drugs, impacting their absorption, activation, and elimination. Probiotics, by reversing the dysbiosis, can help regulate these interactions, optimize drug responses, and minimize adverse effects. Previous studies have shown that the gut microbiome can inactivate certain drugs by enzymatic modification, while probiotics may mitigate these effects by modulating enzymatic activities within the microbiota (Verma and Shukla 2013).

By reestablishing a balanced microbiome, probiotics enhance drug bioavailability, advocating their inclusion as an adjunctive therapy in CRC. Moreover, probiotics not only help suppress the growth of pathogenic bacteria but also increase the abundance of butyrate-producing bacteria that support intestinal health. Butyrate, a short-chain fatty acid produced by certain bacteria, has anti-inflammatory and anti-carcinogenic properties, that can cause a reduction in tumor cell proliferation and increase apoptosis (Hibberd et al. 2017).

Furthermore, probiotics such as *Lactobacillus* and *Bifidobacterium*, can inhibit the activation of carcinogens by reducing enzymes like β-glucuronidase and nitroreductase, which are involved in the metabolism of pro-carcinogenic substances (Verma and Shukla 2013). Probiotic administration has also been shown to enhance gut barrier integrity, minimize systemic inflammation, and limit the spread of pathogenic bacteria that may contribute to tumorigenesis (Wang et al. 2021).

The immunomodulatory effects of probiotics support their potential use as adjunctive therapy in CRC treatment. By interacting with the immune system through toll-like receptors (TLRs), particularly TLR4, probiotics help modulate the host’s immune response to cancer. Studies have shown that probiotics can inhibit the expression of TLR4 and COX-2, reducing inflammation and promoting an anti-tumor response (Chong 2014). Probiotics also activate dendritic cells, which then mature and release cytokines such as IL-12, subsequently activating natural killer (NK) cells. This activation enhances the production of IFN-γ and granulocyte-macrophage colony-stimulating factor (GM-CSF), which is crucial in producing an anti-tumorigenic immune response (Raman et al. 2013). Probiotics release antimicrobials and improve intestinal permeability, tight junction function, and enzyme activity in patients with colorectal cancer (Sivamaruthi et al. 2020).

Specifically, *Lactobacillus fermentum* can prevent NF-κB signaling and restrict cell development causing apoptosis in colorectal cell lines (Alam 2022). While *Lactobacillus delbrueckii* can effectively suppress proliferation and trigger apoptosis via the caspase 3-dependent pathway in colon cancer cells (Wan et al. 2014).

Drug repurposing, using existing medications to combat new medical conditions, is a revolutionary method that addresses the challenges of drug development and healthcare issues (Kulkarni et al. 2023; Oprea and Mestres 2012). Telmisartan is an Angiotensin-II Type 1 Receptor (AT1R) blocker that is used primarily in hypertension. In addition to its initial use, it has been shown to have potential anti-cancer properties by suppressing cell growth and apoptosis in colon cancer cells (Lee et al. 2014). Significant evidence has been shown that AT1R expression is associated with enhanced angiogenesis and cellular proliferation rate (Arrieta et al. 2015). In a recent study, the hypertension medication telmisartan was used as an adjuvant treatment for breast cancer (Kumar et al. 2023).

The importance of biomarkers in the early diagnosis, management, and follow-up of CRC cannot be overstated. Interleukin 6 (IL-6), an inflammatory cytokine, has been implicated in developing tumors and various inflammatory diseases (Kumari et al. 2016). Carcinoembryonic antigen (CEA) is a biomarker often elevated in CRC and can be used to monitor disease development and treatment efficacy (Hall et al. 2019). Moreover, angiogenesis is associated with vascular endothelial growth factor (VEGF) level, which indicates tumor growth and metastasis (Melincovici et al. 2018). In addition to these protein indicators, bacterial biomarkers such as *Lactobacillus*,* Bacteroides*,* Clostridium*, and *Ruminococcus* are important indicators to monitor CRC development. The relative abundance of these taxa varies between CRC patients and healthy individuals, hence, it can be potentially used for non-invasive CRC screening and personalized therapeutic strategies (Rinninella et al. 2019). The combination of *Lactobacillus* spp. and telmisartan has not been previously tested in CRC treatment. This study aimed to assess the effects of a commercial *Lactobacillus* spp. mixture and telmisartan on a CRC-afflicted rat model, either singly or in combination. Protein markers, IL-6, VEGF, and CEA were evaluated in different groups within the experimental model to assess the potential therapeutic impact of *Lactobacillus* spp. and telmisartan. The relative abundance of *Bacteroides* spp., *Clostridium* spp., *Lactobacillus* spp., *Ruminococcus* spp., and *Clostridium coccoides* was measured by real-time PCR to determine changes in the microbial composition and CRC development.

## Materials and methods

### Experimental animals

Seventy male Sprague-Dawley rats weighing 200 and 250 g were purchased from Delta University for Science and Technology in Egypt. Animals with significant baseline health issues were excluded from the study. The rats underwent a two-week acclimatization phase to adapt to their new environment, and standardized conditions were maintained throughout the experiment with a temperature of 25 ± 2 °C and relative humidity of 65 ± 5%. The rats had unrestricted access to water and standard chow and were housed in polypropylene cages with five rats per cage within the animal facility for the study’s duration. The sample size was calculated based on power analysis using a significance level of 0.05 and a power of 0.8. The effect size was estimated from preliminary data. The experimental procedures were reviewed and approved by the Research Ethics Committee of the Faculty of Pharmacy, Cairo University, Egypt (Protocol No. MI 2967).

### Induction of colorectal cancer in rats

After the acclimatization phase, the rats were randomly assigned to receive subcutaneous injections of 1,2-dimethylhydrazine (DMH, Sigma-Aldrich, St. Louis, MO, USA). DMH was prepared in a 0.9% saline solution. The rats were administered a weekly dose of 40 mg/kg body weight for eight weeks (Salim et al. 2020).

### Preparation and administration of the probiotic suspension

Sachets of the commercial preparation “Lacteol fort,” which contain 200 mg of *Lactobacillus* LB, a blend of *Lactobacillus delbrueckii* and *Lactobacillus fermentum*, each providing 5 billion colony-forming units (CFU)/sachet of probiotics were used. Lacteol fort is produced by the 10th of Ramadan for Pharmaceutical Diagnostic Reagents (Rameda) in the 6th of October City under a license from Aptalis Pharma SAS - France. The sachets were dissolved in distilled water, and the concentration was carefully adjusted so that a 0.5 mL suspension contained 2.7 × 10^8^ colony-forming units (CFU) per milliliter. The prepared suspension was administered orally to the rats via gastric intubation (Fooladi et al. 2015).

### Telmisartan preparation and route of administration

Telmisartan was purchased from Sigma-Aldrich (St. Louis, MO, USA) and suspended in 0.5% carboxymethyl Cellulose solution (El Nasr Pharmaceutical Chemicals Co., Cairo, Egypt). The rats were orally administered 10 mg/kg body weight of telmisartan solution in a gastric tube (Zhou et al. 2022).

### The experimental design

**Fig. 1:**
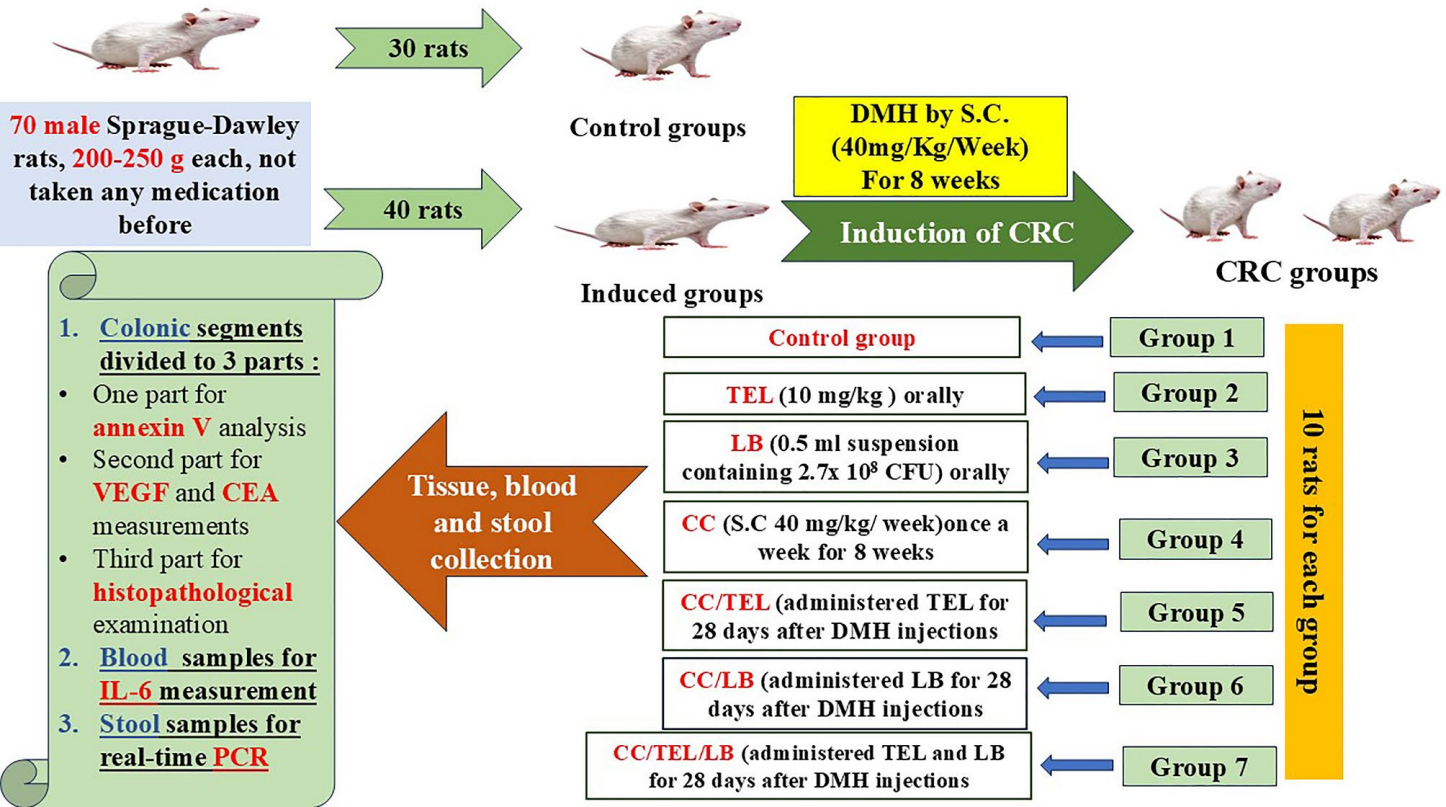
Experimental design diagram

The rats were fed a standard diet and distilled water ad libitum as previously described, during the 84 days of the experiment. They were randomly divided into seven groups (*n* = 10) where randomization was carried out using a computer-generated random number table before the start of the experiment: G1 (Control Group), the rats were administered phosphate-buffered saline (PBS) and a standard diet; G2 (TEL Group), the rats received daily 10 mg/kg telmisartan PO; G3 (LB Group), the rats were administered daily 0.5 mL suspension of *Lactobacillus* spp. containing 2.7 × 10^8^ CFU PO by oral gastric tube; G4 (CC Group) the rats were subcutaneously injected weekly with 40 mg/kg body weight DMH for eight weeks; G5 (CC/TEL Group) the rats were orally administered telmisartan daily for 28 days following eight weeks of DMH injections; G6 (CC/LB Group) the rats were administered *Lactobacillus* spp. suspension for 28 days following eight weeks of DMH injections; and G7 (CC/TEL/LB Group) the rats received DMH for eight weeks, followed by the administration of both *Lactobacillus* spp. suspension and telmisartan for 28 days (Fig. 1**)**.

The dosage regimen of the treatment groups was selected based on pilot experiments that confirmed its efficacy. At the end of the experiment, the rats were anesthetized with thiopental sodium (20 mg/kg) and subsequently sacrificed by decapitation. Blinding was implemented during data collection and analysis. The researchers performing the assessments were unaware of the group allocations to reduce potential bias.

### Sample collection and analysis

Colonic segments were removed from each rat, and the fatty tissue was washed off in cold, normal saline. The segments were then divided into three parts for various analyses. One part was processed for the analysis of Annexin V expression by flow cytometry, while the second part was used in ELISA for the measurement of VEGF and CEA levels. Finally, the third part was fixed in 10% neutral buffered formalin and stained with hematoxylin and eosin for histopathological examination.

Blood samples were drawn from the retro-orbital plexus while the rats were under anesthesia, using heparinized capillary tubes before decapitation. Following coagulation, the sera were separated immediately by centrifugation at 4000 rpm for 10 min and analyzed to estimate IL-6 levels. Stool samples were collected and kept at − 80 °C until DNA extraction for conventional and real-time PCR analyses.

### Detection of apoptosis by measurement of annexin V level

Apoptosis was evaluated using the Annexin V-FITC Apoptosis Detection Kit (Abcam, ab14085, Cambridge, UK) according to the manufacturer’s guidelines. Briefly, the cells were collected and counted. A suspension of 1 × 10^5^ cells was prepared, and 500 µL of Annexin V binding buffer, 5 µL of Annexin V-FITC, and 5 µL of propidium iodide (PI) were added to the suspension. This mixture was incubated in the dark at room temperature for 5 minutes. After incubation, the samples were analyzed by flow cytometry using a BD Accuri™ C6 Plus Flow Cytometer (BD Biosciences, New Jersey, USA). The flow cytometer was set to excite at 488 nm and detect emission at 530 nm to evaluate FITC signals, while PI staining was assessed with a phycoerythrin emission signal detector.

### Assessment of inflammatory mediators and biomarkers

The inflammatory mediator IL-6 was quantified in serum samples using a commercial ELISA kit (Catalog Number: CSB-E04640r) from Cusabio, Houston, USA, according to the manufacturer’s instructions. CEA levels were assessed in tissue homogenates using an ELISA kit (Catalog Number: CSB-E13926r) from Cusabio, Houston, USA, following the provided protocol. VEGF was measured in tissue homogenates using a Rat VEGF Quantikine ELISA kit (Catalog Number: RRV00, R&D Systems, Minneapolis, USA, following the manufacturer’s guidelines.

### Histopathological preparation and examination of the colon specimens

Rectal and distal colon specimens were collected from each rat and given a coding number so that the specimens could be analyzed blindly in the pathology lab. The collected specimens underwent fixation in 4% formalin and embedded in paraffin. A pathologist, blinded to the experimental groups, performed standard histological analyses on the samples. The tissue specimens underwent dehydration through a series of alcohol concentrations, followed by embedding in paraffin. Sections of 4–5 μm thickness were prepared using a microtome. After deparaffinization and rehydration, these sections were stained with hematoxylin and eosin (H&E) for histopathological evaluation (Saber et al. 2019).

### Detection of the selected gut microbiota by PCR

According to the manufacturer’s instructions, DNA was extracted from stool samples using the QIAamp DNA Stool Mini Kit (Qiagen Inc., Hilden, Germany). DNA extract was quantified using a NanoDrop spectrophotometer (Jenway Nano, UK) to ascertain DNA concentration and purity then stored at -20 °C until use. The extracted DNA was amplified in a thermal cycler (Prime thermal cycler, UK) in a 25 µL reaction mixture. The reaction mixture included 2.5 µL of DNA template, 12.5 µL of MyTaq Red Mix (Bioline Co., UK), 1 µL of each forward and reverse primer (Table 1), and nuclease-free water to reach a final volume of 25 µL. The PCR conditions were as follows: an initial denaturation step at 94 °C for 5 min, followed by 35 cycles of denaturation at 94 °C for 30 s, annealing at the specific temperature for each primer pair (Table 1) for 30 s, and extension at 72 °C for 30 s, with a final extension at 72 °C for 3 min. The amplified PCR products, along with a GeneRuler 100 bp Plus DNA Ladder (Thermo Scientific, USA), were separated on a 1.5% agarose gel. The gel was stained with ethidium bromide, visualized under a UV transilluminator, and photographed (Gel documentation system GELDOC-IT^2^ IMAGER, Cambridge, UK) (Wong et al. 2017). Positive and negative controls were used to ensure the accuracy and reliability of the PCR.

**Table 1:** List of primers, their sequences, annealing temperatures, and amplicon sizes used to identify the selected distinct bacterial species

Primer name	Primers	Primer sequence	Annealing (Tm, °C)	Size (bp)	Reference
Universal Primers	F R	GAGTTTGATCCTGGCTCAG GCTGCCTCCCGTAGGAGT	51	312	Ginige et al. (2013)
*Bacteroides* spp.	F R	AAGGGAGCGTAGATGGATGTTTA CGAGCCTCAATGTCAGTTGC	55	193	Huijsdens et al. (2002)
*Clostridium* spp.	F R	CGGTACCTGACTAAGAAGC AGTTTGATTCTTGCGAACG	50	429	Vemuri et al. (2018)
*Clostridium coccoides*	F R	AAATGACGGTACCTGACTAA CTTTGAGTTTCATTCTTGCGAA	50	440	Matsuki et al. (2002)
*Ruminococcus* spp.	F R	GGCGGCCTACTGGGCTTT CCAGGTGGATAACTTATTGTGTTAA	60	157	Wu et al. (2019a, b, 2022)
*Lactobacillus* spp.	F R	AGCAGTAGGGAATCTTCCA CACCGCTACACATGGAG	50	334	Collado et al. (2008)

### Quantitative PCR of the selected microbial markers

The relative abundance of *Bacteroides* spp., *Clostridium* spp., *Lactobacillus* spp., *Ruminococcus* spp., and *Clostridium coccoides* was quantified using quantitative PCR (qPCR), with the 16 S rRNA gene serving as a housekeeping reference. The qPCR reactions were conducted in a total volume of 20 µL. Each reaction consisted of 10 µL of SYBR Green with low ROX (Enzynomics, Daejeon, Korea), 1 µL of forward primer (10 µM), 1 µL of reverse primer (10 µM), 6 µL of nuclease-free water, and 2 µL of DNA template.

The qPCR was performed under the following cycling conditions: an initial denaturation at 95 °C for 5 min, followed by 45 cycles of denaturation at 95 °C for 20 s, annealing at the temperature specified in Table 1 for 20 s, and extension at 72 °C for 40 s. All qPCR assays were performed on a QuantStudio 5 Real-Time PCR System (Thermo Fisher Scientific, Waltham, MA, USA). Cycle threshold (Ct) values were determined using the QuantStudio 5 software. Amplification specificity was confirmed by melting curve analysis. The relative abundance of the target microbial markers was calculated as normalized units relative to the total bacterial count in each sample using the 2^−ΔCt^ method (Wong et al. 2017).

where: ΔCt = average Ct value of each target marker - average Ct value of total bacteria.

### Statistical analysis

The data was evaluated using Tukey’s post-hoc test and one-way analysis of variance (ANOVA). The histopathological scores were analyzed using Dunn’s multiple comparison post-test and the non-parametric Kruskal-Wallis test. Means and standard error of the mean (SEM) were used to represent the data. A *p*-value of less than 0.05 was considered significant. The GraphPad Prism software version 9.0 (GraphPad Software Inc., CA, USA) was used to analyze and represent the data.

## Results

### Effect of TEL and LB on cell apoptosis in CRC

**Fig. 2:**
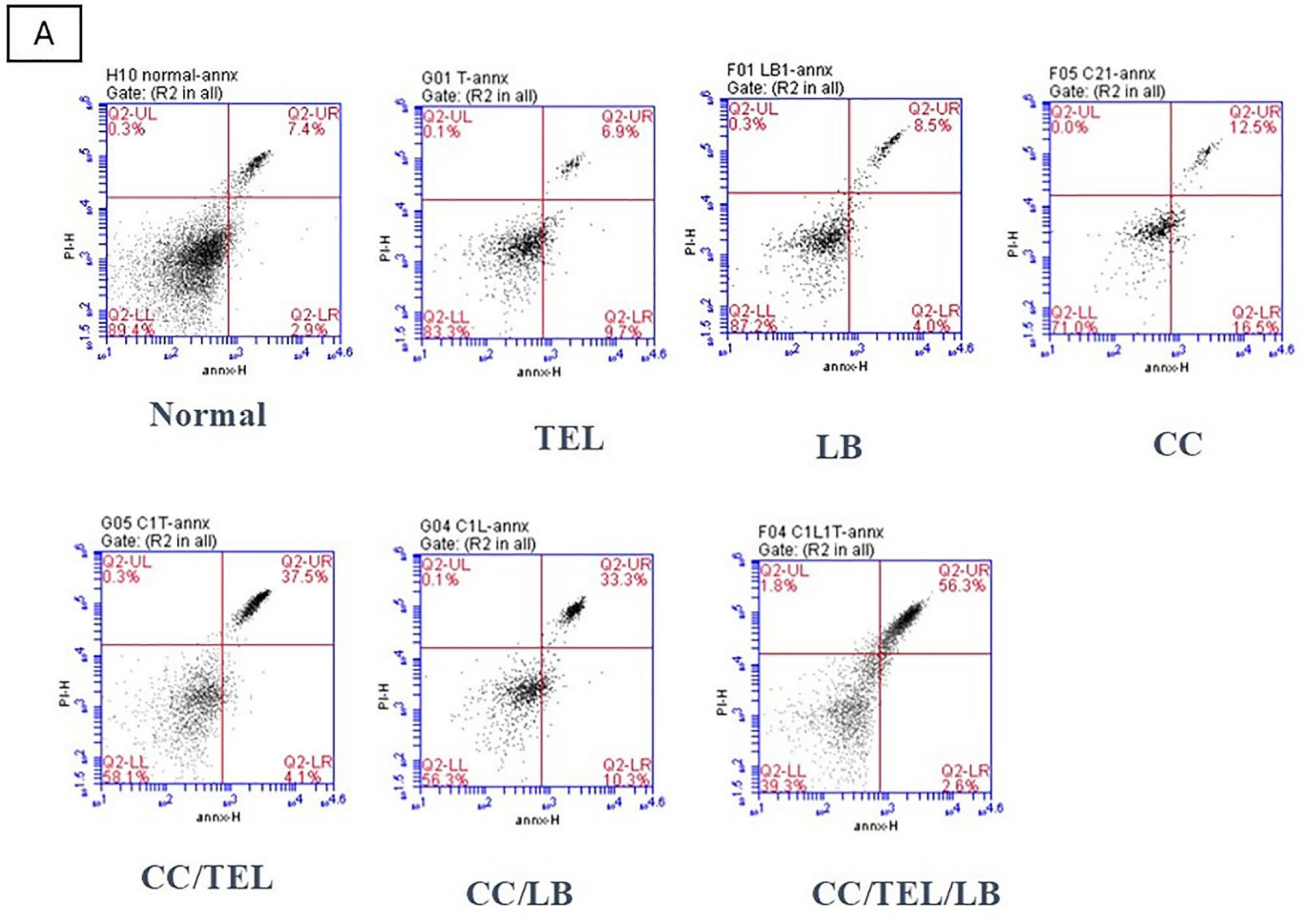

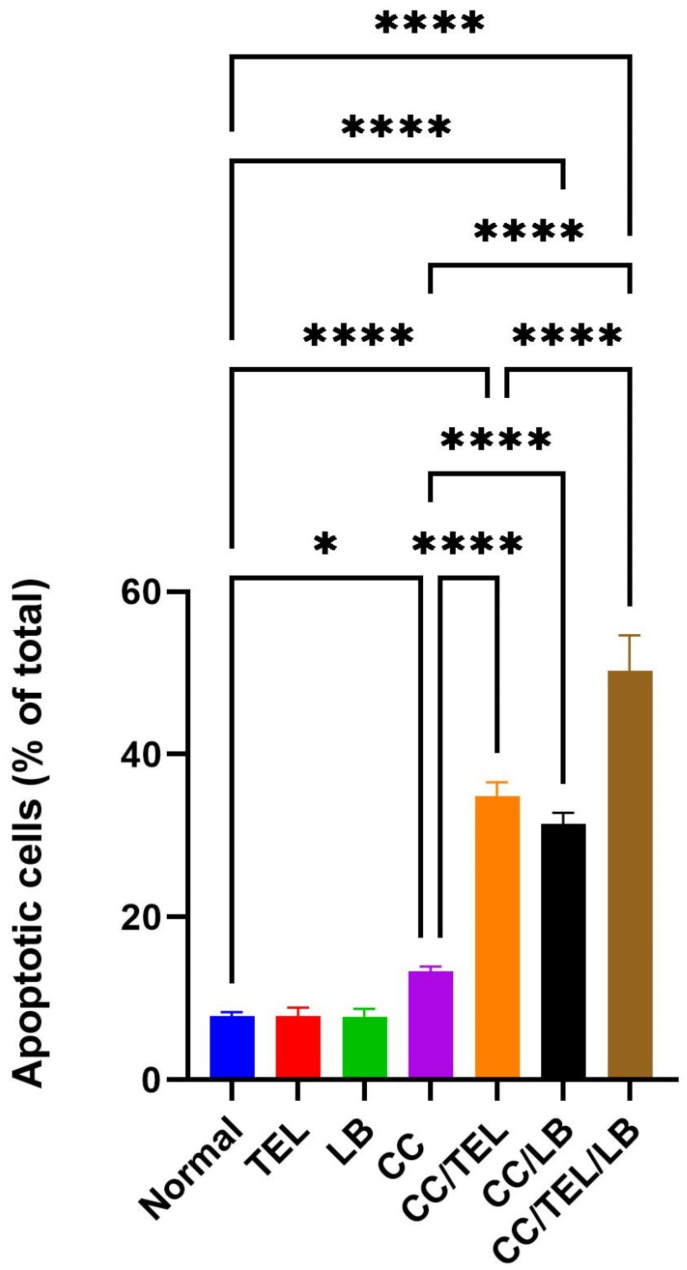
Detection of apoptosis using flow cytometry after staining with Annexin V-FITC/propidium iodide (PI) in the seven groups of the experimental model: Normal: control group, TEL: telmisartan group, LB: *Lactobacillus* mixture group, CC: CRC-induced group, CC/TEL: CRC-induced treated with telmisartan group, CC/LB: CRC-induced treated with *Lactobacillus* mixture group; CC/TEL/LB: CRC-induced treated with telmisartan and *Lactobacillus* mixture group. A: Representative scatter plots displaying the relationship between PI (y-axis) and Annexin V (x-axis). In these plots, viable cells are located in the lower left quadrant, early apoptotic cells in the lower right quadrant, late apoptotic cells in the upper right quadrant, and non-viable necrotic cells in the upper left quadrant. B: Percentage of late apoptotic cells where: *significant difference

An increase in apoptotic cells was detected in the CC group (*p* < 0.05) compared to the control group. There was a marked increase in apoptotic cells in the CC/TEL group (*p* < 0.0001), the CC/LB group (*p* < 0.0001), and the CC/TEL/LB group (*p* < 0.0001) compared to the CC group. Additionally, the level of apoptosis in CC/TEL/LB was significantly higher than that in the CC/TEL group (*p* < 0.0001) (Fig. 2).

### Effect of TEL and LB on vascular endothelial growth factor in CRC

**Fig. 3:**
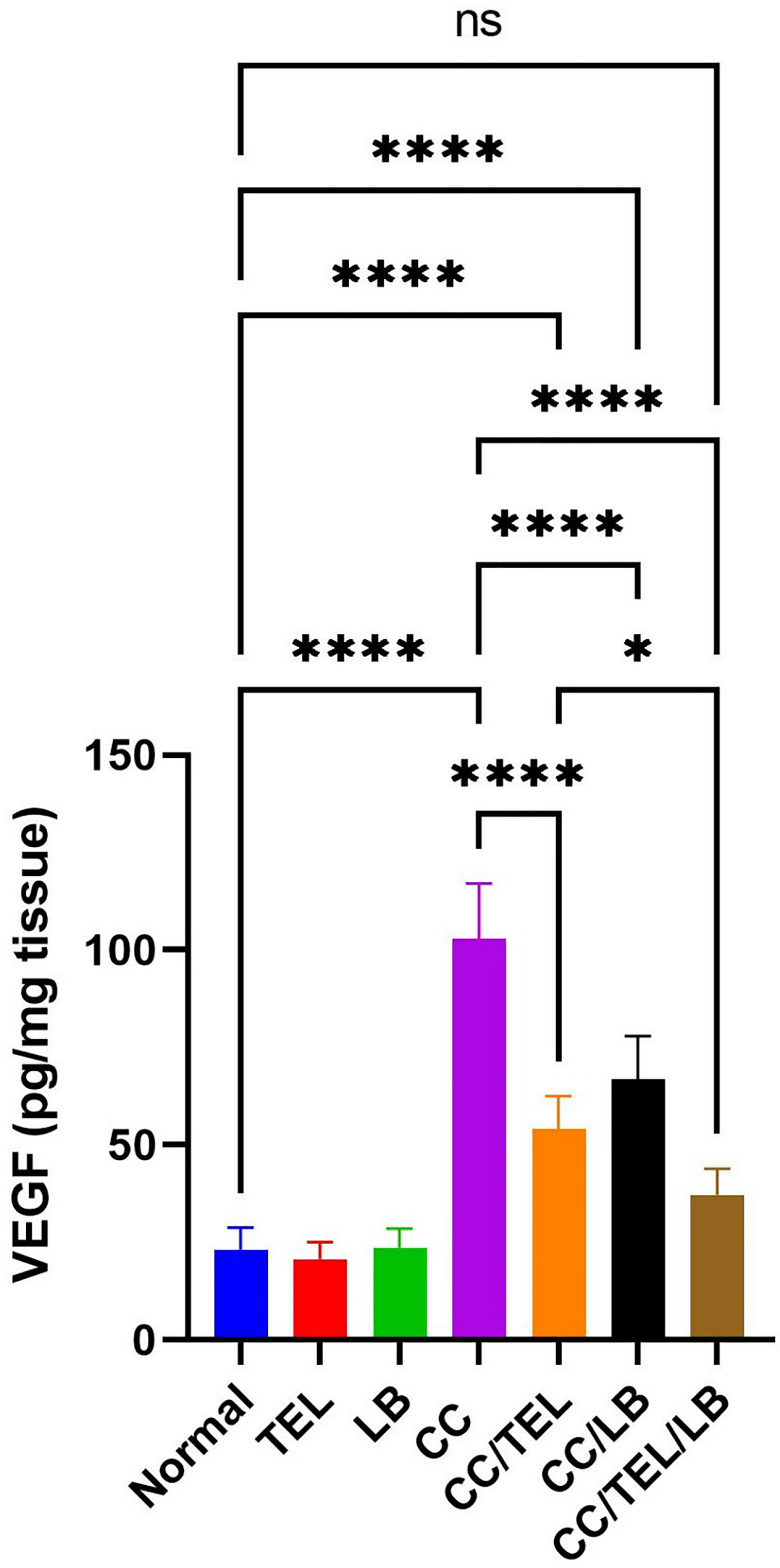
Detection of VEFG levels by ELISA in the seven groups of the experimental model: Normal: control group, TEL: telmisartan group, LB: *Lactobacillus* mixture group, CC: CRC-induced group, CC/TEL: CRC-induced treated with telmisartan group, CC/LB: CRC-induced treated with *Lactobacillus* mixture group; CC/TEL/LB: CRC-induced treated with telmisartan and *Lactobacillus* mixture group. where: pg/ml: picogram per milligram; ns: non-significant and *significant difference

Compared to the control group, VEGF showed a significant difference in the CC group (*p* < 0.0001) (Fig. 3). There was a significant difference in VEGF in the CC/TEL group (*p* < 0.0001), the CC/LB group (*p* < 0.0001), and the CC/TEL/LB group (*p* < 0.0001) compared to the CC group. In addition, there was a significant difference in the CC/TEL/LB group relative to the CC/TEL group (*p* < 0.05) (Fig. 3).

### Effect of TEL and LB on carcinoembryonic antigen level in CRC

**Fig. 4:**
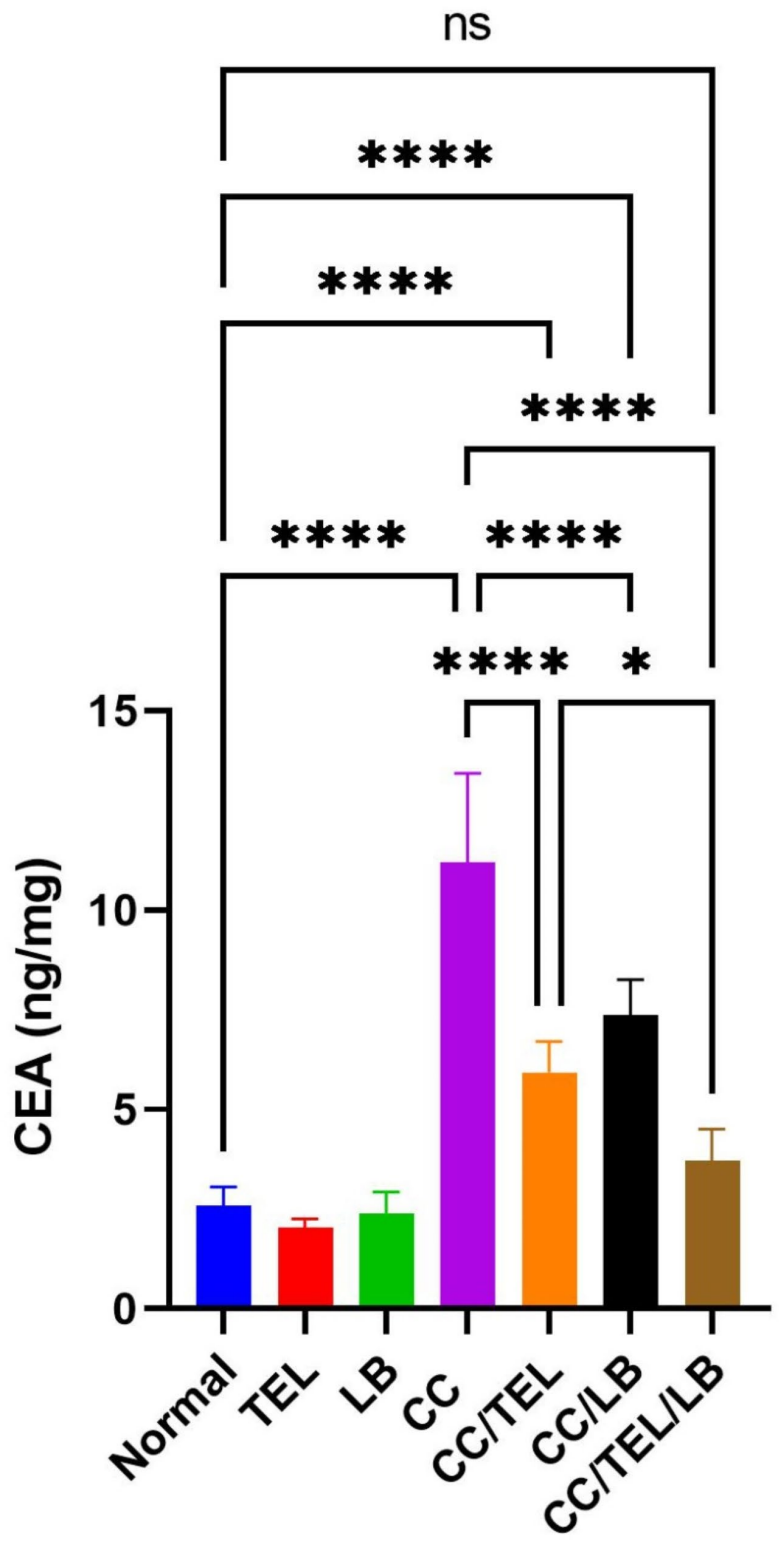
Detection of CEA levels by ELISA in the seven groups of the experimental model: Normal: control group, TEL: telmisartan group, LB: *Lactobacillus* mixture group, CC: CRC-induced group, CC/TEL: CRC-induced treated with telmisartan group, CC/LB: CRC-induced treated with *Lactobacillus* mixture group; CC/TEL/LB: CRC-induced treated with telmisartan and *Lactobacillus* mixture group, where: ng/mg: nanogram per milligram; ns: non-significant and *significant difference

Carcinoembryonic antigen levels (CEA) significantly increased in the CC group compared to the control group (*p* < 0.0001) (Fig. 4). Additionally, there was a significant decrease in CEA in the CC/TEL group (*p* < 0.0001), CC/LB group (*p* < 0.0001), and CC/TEL/LB group (*p* < 0.0001) relative to the CC group. However, there was a significant decrease in the CEA levels in the CC/TEL/LB group relative to the CC/TEL group (*p* < 0.05) (Fig. 4).

### Histopathological examination of colonic specimens

**Fig. 5:**
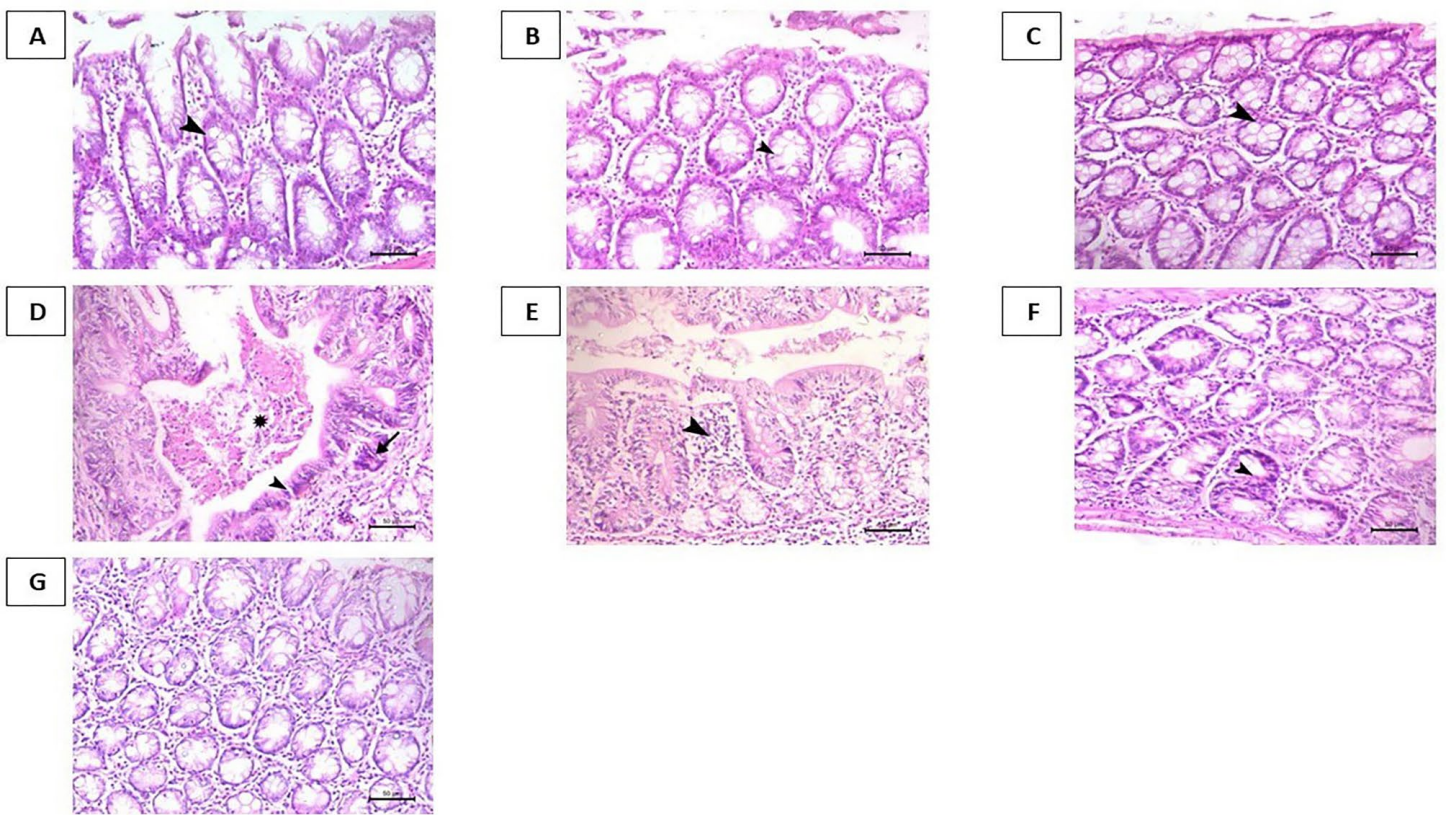
Representative photomicrographs of H&E-stained colon specimens. **A**: Control group showing normal colon mucosa with normal intestinal crypts containing normal goblet cells (arrowhead). **B**: TEL group showing normal colonic mucosa (arrowhead indicates normal goblet cells). **C**: LB group showing normal mucosal lining with normal intestinal glands (arrowhead). **D**: CC group showing early differentiated cystic adenocarcinoma associated with epithelial invasion within the connective tissue stroma (arrow), basal epithelial basophilic hyperplastic changes within the lining epithelium denudation (arrowhead), and formation of necrotic core (asterisk). **E**: CC/TEL group showing decreased basophilic hyperplastic changes and focal mononuclear cell infiltration (arrowhead). **F**: CC/LB group showing focal dysplastic changes within individual mucosal glands (arrowhead). **G**: CC/TEL/LB group showing a marked decrease in intestinal dysplasia and inflammation within the colon mucosa. Scale bar = 50 μm

In the control group, the colonic mucosa displayed a well-organized epithelial lining with regularly spaced crypts of Lieberkühn (Fig. 5A). The arrowhead denotes the presence of abundant goblet cells, characterized by their mucin-rich cytoplasm, which appears to be evenly distributed within the crypts (Fig. 5A). The colonic mucosa from the TEL group showed normal architecture with well-defined crypts (Fig. 5B). The arrowhead points to the goblet cells, which maintain their typical morphology and distribution (Fig. 5B). In the LB group, a well-preserved mucosal lining with intact intestinal glands (arrowhead) was depicted, indicative of healthy tissue morphology (Fig. 5C).

In contrast, in the CC group, severe dysplastic changes, nuclear disorganization, basophilia, epithelial invasion into the connective stroma, and inflammatory cell infiltration were pronounced (Fig. 5D). In the CC/TEL group, the colonic mucosa showed decreased crypt dysplasia with reduced basophilic hyperplastic changes and focal mononuclear cell infiltration was seen (Fig. 5E). The colonic mucosa from the CC/LB group displayed focal dysplastic changes localized within the individual mucosal glands (Fig. 5F). The colonic tissue from the CC/TEL/LB group demonstrated a marked attenuation of intestinal dysplasia and inflammation within the mucosal lining (Fig. 5G).

### Effect of TEL and LB on IL-6 levels in colorectal cancer induced by DMH

**Fig. 6:**
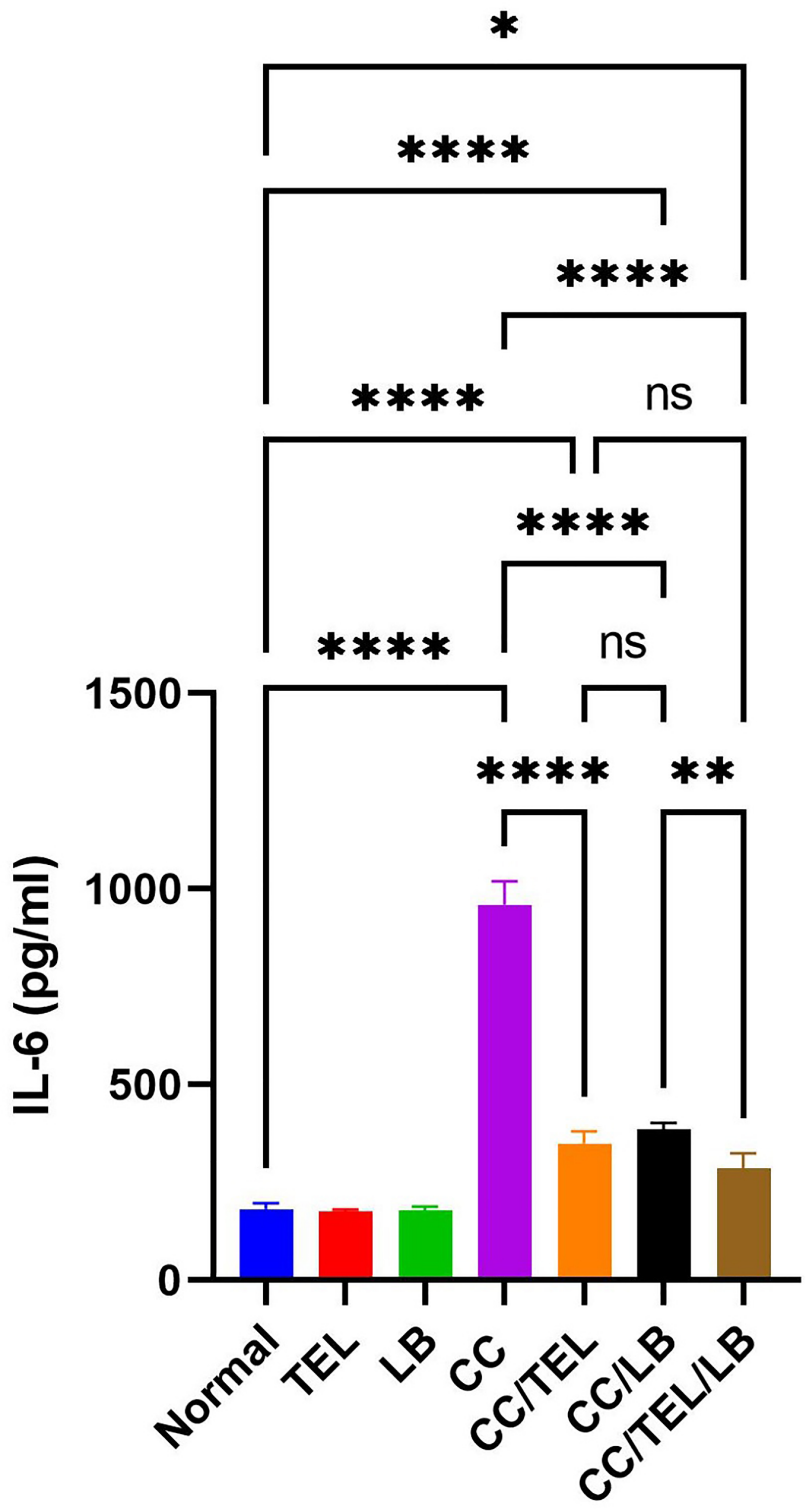
Detection of Interleukin 6 levels by ELISA in the seven groups of the experimental model: Normal: control group, TEL: telmisartan group, LB: *Lactobacillus* mixture group, CC: CRC-induced group, CC/TEL: CRC-induced treated with telmisartan group, CC/LB: CRC-induced treated with *Lactobacillus* mixture group; CC/TEL/LB: CRC-induced treated with telmisartan and *Lactobacillus* mixture group, where: pg/ml: picogram per milliliter; ns: non-significant and *significant difference

The levels of IL-6 significantly increased in the CC group compared to the control group (*p* < 0.0001). There was a significant decrease in IL-6 levels in the CC/TEL group (*p* < 0.0001), CC/LB group (*p* < 0.0001), and CC/TEL/LB group (*p* < 0.0001) compared to the CC group. There was no significant difference in the IL-6 level of the CC/TEL/LB group compared with the CC/TEL group (Fig. 6).

### Detection of the target bacterial markers by PCR

By standard uniplex PCR, the targeted five bacterial genera associated with colorectal cancer (CRC) were detected in all the groups of the experimental model including the control group, DMH-treated group, LB-treated group, and TEL-treated group.

### Quantification of the selected bacterial markers by real-time PCR

**Fig. 7:**
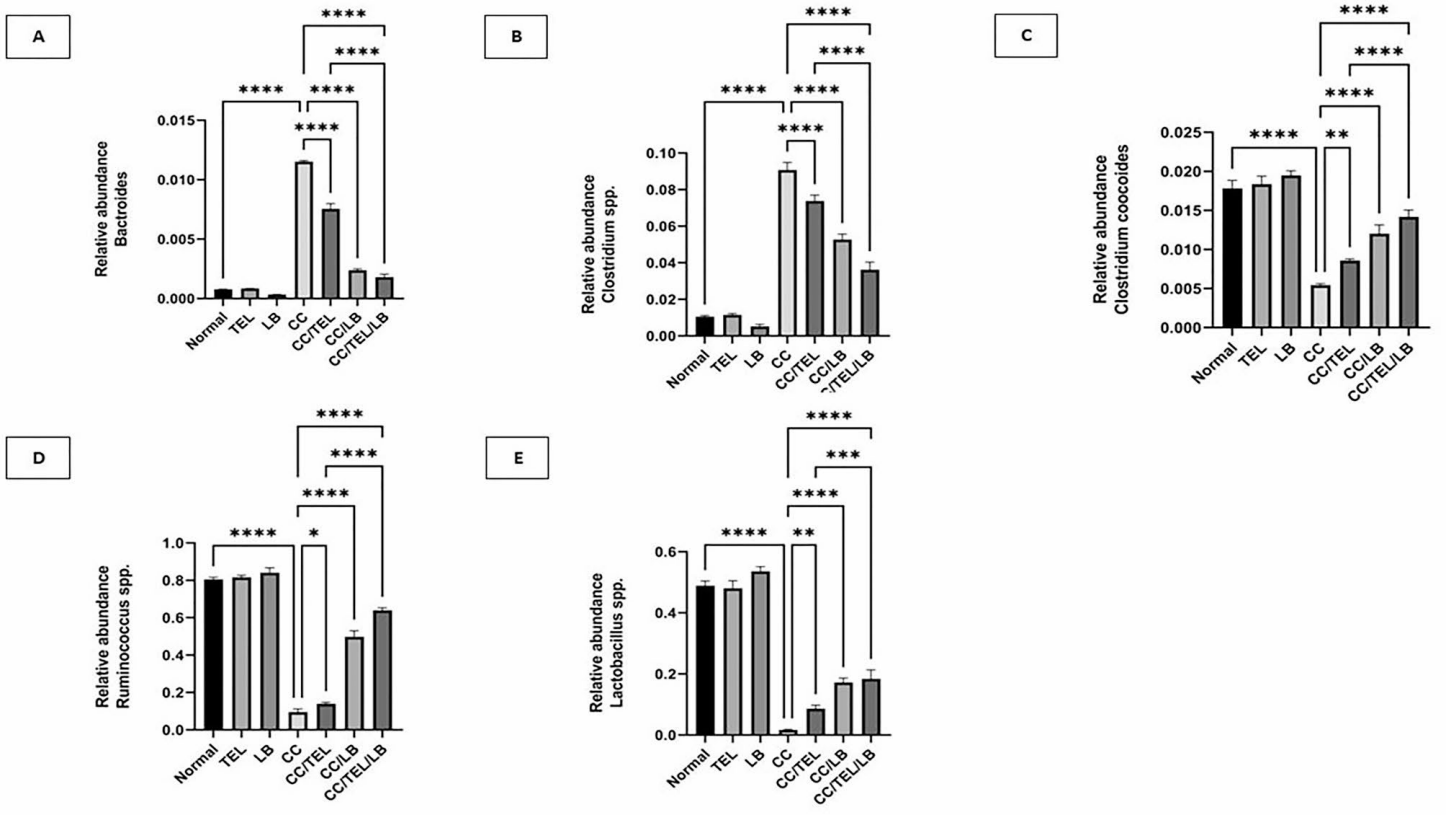
The relative abundance of the selected five bacterial markers: **A**: *Bacteroides* spp, **B**: *Clostridium* spp. **C**: *Clostridium coccoides*, **D**: *Ruminococcus* spp., **E**: *Lactobacillus* spp.; measured by qPCR in the seven groups of the experimental model: Normal: control group, TEL: telmisartan group, LB: *Lactobacillus* mixture group, CC: CRC-induced group, CC/TEL: CRC-induced treated with telmisartan group, CC/LB: CRC-induced treated with *Lactobacillus* mixture group; CC/TEL/LB: CRC-induced treated with telmisartan and *Lactobacillus* mixture group where ns: non-significant and *significant difference

The abundance of the five bacterial markers (*Bacteroides* spp., *Clostridium* spp., *Clostridium coccoides*,* Ruminococcus* spp., and *Lactobacillus* spp.) in the different experimental groups was expressed relative to the total bacterial count measured using universal primers. In the CC group, the relative abundance of *Bacteroides* spp. and *Clostridium* spp. was higher than that in the control and treated groups. Conversely, *Clostridium coccoides*,* Ruminococcus* spp., and *Lactobacillus* spp. were found to be more abundant in the control and treated groups than in the CC group (Fig. 7).

The CC group showed a significant increase in *Bacteroides* spp. abundance relative to the control group, with a statistical significance of *p* < 0.0001. When comparing the CC group to the treatment groups (CC/TEL, CC/LB, and CC/TEL/LB), all the groups showed a significant decrease in *Bacteroides* abundance. The combination group CC/TEL/LB showed a significantly lower level of *Bacteroides* when compared to CC/TEL (Fig. 7A).

No significant difference in the relative abundance of *Clostridium* spp. was detected in the control group relative to the TEL-group or LB group under normal conditions. However, a marked reduction in *Clostridium* spp. abundance was observed when comparing the CC group with the treatment groups (CC/TEL, CC/LB, and CC/TEL/LB). In addition, a significant decline of *Clostridium* spp. in CC/TEL/LB was detected when compared to CC/TEL (Fig. 7B).

The relative abundance of *Clostridium coccoides* in the control group was not significantly different in the TEL and LB groups. The CC group showed a reduced relative abundance of *Clostridium coccoides* significantly when compared to the control group. When comparing the CC group to the treatment groups (CC/TEL, CC/LB, and CC/TEL/LB), there was a significant increase in *Clostridium coccoides*. Moreover, the CC/TEL/LB group showed an increase in *Clostridium coccoides* relative to the CC/TEL group (Fig. 7C).

No significant difference in the relative abundance of *Ruminococcus* spp. was observed between the control group and the TEL group or LB-treated group. A significant decrease in the relative abundance of *Ruminococcus* spp. was observed in the CC group compared to the control group (*p* < 0.0001). The treatment groups (CC/TEL, CC/LB, and CC/TEL/LB) showed a significant increase in the relative abundance of *Ruminococcus* spp. compared to the CC group. The combination CC/TEL/LB group showed a higher level of *Ruminococcus* spp. compared to the CC/TEL group (Fig. 7D).

TEL group and LB group showed similar *Lactobacillus* spp. abundance levels compared to the control group, with no statistically significant difference. The CC group exhibited a significantly lower *Lactobacillus* spp. abundance compared to the control group (*p* < 0.0001). The *Lactobacillus* spp. abundance in the treatment groups (CC/TEL, CC/LB, and CC/TEL/LB) was significantly higher than in the CC group. The CC/TEL/LB group showed an even higher level in *Lactobacillus* spp. abundance compared to the CC/TEL group (Fig. 7E).

## Discussion

In this work, the combination of telmisartan and *Lactobacillus* species exerted an enhanced effect in the treatment of CRC through the assessment of selected biomarkers, target gut microbiota composition, and histopathological examination of colonic specimens. The effect of probiotics on apoptosis in CRC was investigated. Apoptosis is an important process that results in a programmed cell death necessary for maintaining cellular homeostasis and inhibiting tumorigenesis (Ding et al. 2020). In cancer cells, apoptosis is often dysregulated, this leads to cell death evasion and uncontrollable proliferation (Dandoti 2021).

Promoting apoptosis in cancer cells has emerged as a promising therapeutic strategy in cancer treatment. Interestingly, in our study, a significant increase in apoptosis was observed in the combined telmisartan-probiotic-treated group compared to the control groups. Previous work reported the apoptotic-inducing effects of probiotics, such as *Lactobacillus acidophilus* and *Lactobacillus rhamnosus* and *Bifidobacterium*, in various cancer models, including CRC (Eslami et al. 2019; Leung et al. 2024). For example, *Lactobacillus* species can modulate essential regulatory proteins involved with cell survival and proliferation pathways, thereby stimulating apoptosis in cancer cells (Nowak et al. 2019). Moreover, the induction of apoptosis by probiotics has been suggested as a mechanism for their potential anti-tumorigenic properties, providing a physiological means to surveil and eliminate dysplastic cells in the colon (Sadrekarimi et al. 2022). The present study shed light on the critical roles of IL-6, VEGF, and CEA in CRC progression and response to therapy by detecting the change in their levels. Rats with CRC had higher levels of the selected biomarkers, indicating that tumoral activity, neoangiogenesis, and inflammation all played a role in the process. Whereas the combined treatment (CC/TEL/LB) group had decreased levels of these biomarkers proving the positive therapeutic effect of the tested drugs.

VEGF is a key player in triggering angiogenesis, a crucial step in tumor growth and metastasis (Melincovici et al. 2018).VEGF levels significantly increased in the CRC group, suggesting that it is responsible for increasing the tumor blood supply and supporting tumor progression. In contrast, levels were reduced in the treatment groups, which implies that antiangiogenic mechanisms could be used to prevent tumor growth in CRC. Detecting VEGF levels can aid in the early detection of disease relapse and the selection of patients for anti-angiogenic treatments, not just for tumor progression.

CEA is a known tumor indicator for CRC, and it is particularly elevated in advanced disease (Wu et al. 2022). Increased CEA levels within the CC group were detected, rendering this marker a tool for monitoring the disease severity and progression. As noted in the treatment (CC/TEL/LB) group, the responsiveness of CEA levels further demonstrates its clinical role in monitoring therapeutic response, thereby reinforcing CEA as both a prognostic indicator and a marker for treatment outcome.

In this work, IL-6, an inflammatory cytokine, was significantly higher in the CC group, indicating a systemic inflammatory response. This response is usually accompanied by tumor progression in CRC patients (Mager et al. 2016). A decline in the levels of IL-6 among the treated groups reflects a decreased inflammation level beneficial in improving overall survival and disease-free survival. IL-6 can also be used as a diagnostic and prognostic biomarker, as its high level is associated with poor survival outcomes (Xu et al. 2016).

In summary, the increased levels of VEGF, CEA, and IL-6 in the CC group and their decrease in the treated group emphasize their significance in CRC detection, progression, and therapy effectiveness. Therefore, tracking these biomarkers can offer personalized and effective management of CRC.

Functionally, the colonic mucosa is vital for secreting mucus that lubricates and protects the colonic epithelium (Pelaseyed et al. 2014; Quansah et al. 2023). In the control group during the histopathological analysis, the mucosal barrier was intact and functional, in addition, the absence of inflammatory infiltrates and structural distortions suggested a healthy colonic mucosa. In our work, telmisartan administration did not adversely affect the goblet cell population or the general standard histological structure of the colon. However, when telmisartan was administered to the CRC-induced rat model, it effectively alleviated the pathological alterations associated with the disease condition. Additionally, monotherapy by *Lactobacillus* spp. mitigated the dysplastic alterations related to CRC. The dual therapy with telmisartan and *Lactobacillus* spp. showed an enhanced effect and was effective in suppressing the pathological hallmarks of the disease state as detected in the experiment’s CC/TEL/LB group of rats.

*Firmicutes*, *Bacteroides*, *Proteobacteria*, and *Actinobacteria* are the four primary phyla that comprise the human gut microbiota (Ghosh and Pramanik 2021). These bacterial phyla play a vital role in CRC progression (Hugon et al. 2015). Dysbiosis can disrupt the intestinal barrier, which can activate both the innate and adaptive immune systems, resulting in persistent inflammation (Keku et al. 2015).

It has been reported that individuals with CRC have less richness and diversity in their fecal microbiota than healthy individuals (Castellarin et al. 2012; Feng et al. 2015). The gut microbiome has the potential to be a new biomarker for CRC diagnosis and prediction of different stages (Temraz et al. 2019).

In this work, target genera were accurately identified and quantified using conventional and real-time PCR. On one hand, real-time PCR is necessary to measure the abundance of target genera (Chen et al. 2023). On the other hand, 16 S rRNA sequencing of the microbial community is a powerful tool for the analysis of the entire community. However, we chose to focus on the change in the abundance of selected genera known for their role in CRC rather than the entire community. This approach allowed us to efficiently analyze and quantify the target genera in the groups of the experimental model.

Recent studies have determined specific microbial genera as potential biomarkers for several health conditions, including CRC. Mostly, five microbial groups—*Bacteroides* spp., *Clostridium* spp., *Clostridium coccoides*,* Lactobacillus* spp., and *Ruminococcus* spp.—are increasingly related to CRC (Huang et al. 2022). *Bacteroides* spp. and *Clostridium* spp. are involved in several metabolic processes and interactions with the gut microbiome that may affect cancer risk (Chattopadhyay et al. 2022). *Clostridium coccoides* produces short-chain fatty acids such as butyrate, which are beneficial to colon health. Butyrate can potentially prevent colon cancer by inducing apoptosis in cancer cells and its anti-inflammatory properties (Wang et al. 2012). *Ruminococcus* spp. plays a role in fiber digestion and butyrate production, which may prevent CRC by affecting inflammation, cellular health, and microbial balance (O’keefe 2016). It is known that *Lactobacillus* spp. maintains gut health and possesses potential protective effects against cancer (Dempsey and Corr 2022). The relative abundance of these bacteria in the gut can indicate pathological alterations linked to colorectal cancer. Subsequently, the variations of these microbial signatures can be used as diagnostic markers.

Probiotics can be employed in both preventive and therapeutic strategies. Lactic acid produced by some *Lactobacillus* species can affect the colon pH and possibly prevent the degradation of amino acids (Garbacz 2022). *Lactobacillus* species are widely recognized for their antioxidant capabilities, which include regulating the levels of antioxidant enzymes, preventing reactive oxygen species (ROS), and scavenging metals (Mishra et al. 2015; Wang et al. 2017). The enhancement of adhesion to mucosal surfaces has been demonstrated by lactate production by lactobacilli fermentation (Chang et al. 2021).

Our study showed that *Lactobacillus* spp. abundance increased significantly in the control and treated groups but significantly decreased in the diseased group. These results are supported by the fact that *Lactobacillus* spp. has anti-inflammatory and protective properties that can counteract CRC (Borges-Canha et al. 2015; Liu et al. 2021; Zou et al. 2018). The increase in *Lactobacillus spp*. abundance observed in the treated group proves that probiotic supplementation is beneficial in treating colorectal cancer. This is particularly impressive considering that *Lactobacillus* species like *L. fermentum* and *L. delbrueckii* have been known for their ability to withstand acidic environments, adhere to mucosal surfaces, and regulate immune responses (Ann Catherine and Prakash 2016; Archer and Halami 2015). Evidence suggests that *L. fermentum* and *L. delbrueckii* have protective effects against colon cancer. The latter is responsible for the decreased growth of colon cancer cells and apoptosis, which could be attributed to Caspase-3-dependent pathways and suppressed MMP-9 activity (Wan et al. 2014). Furthermore, *L. fermentum* has been proven to prevent mice from developing colon cancer (Asha and Gayathri 2012). The potential for probiotics to help reduce CRC is based on a positive association between higher relative abundance in *Lactobacillus* spp. and a healthier colonic environment.

By adopting a drug repurposing approach, we assessed the therapeutic effectiveness of telmisartan in CRC. Recently, it was reported that telmisartan can affect the gut microbiome in a similar way to probiotic treatments by *Lactobacillus rhamnosus*, leading to a better understanding of its more extensive health effects beyond controlling blood pressure (Robles-Vera et al. 2020a, b; Wu et al. 2019a, b). Telmisartan administration resulted in a reduction in the Firmicutes/Bacteroidetes ratio, a biomarker linked to several diseases (Robles-Vera et al. 2020a, b). Beckmann et al. proposed that telmisartan modulates the gut microbiome, potentially affecting metabolic health, short-chain fatty acids, metabolic endotoxemia, and cannabinoid receptors (Beckmann et al. 2021). In our work, the administration of telmisartan led to a decrease in *Bacteroides* spp. and *Clostridium* spp. abundance with an increase in *Ruminococcus* spp. and *Lactobacillus* spp. abundance relative to the CC group. In addition, probiotics, which have the potential to regulate microbial communities, enhanced the effects of telmisartan on gut microbiota in the combined treatment (CC/TEL/LB). Histopathological examination of the colonic segments of the combination of telmisartan (TEL) and *Lactobacillus* (LB) treated group proved their protective effects on the colon, potentially mitigating the pathological changes associated with colorectal carcinogenesis. The attenuation of dysplasia and inflammation highlights the therapeutic potential of this combination in maintaining mucosal integrity and reducing precancerous lesions. Further investigation of these pathways can pave the way to create novel therapeutic approaches targeting the gut microbiome to manage diseases linked to dysbiosis.

This study provides insightful knowledge about the abundance of specific bacterial genera in CRC relative to healthy conditions. It highlights the potential use of VEGF, CEA, and IL-6 as prognostic indicators for CRC disease progression. Our findings demonstrate that the co-administration of *Lactobacillus* spp. with telmisartan enhances apoptotic activity, exerts anti-angiogenic and anti-inflammatory effects, restores gut microbial balance, improves histopathological outcomes, and reduces tumor biomarkers. These results underscore the therapeutic potential of combining *Lactobacillus* spp. with telmisartan as a novel approach for CRC management. However, a longer follow-up period could provide valuable information on the long-term effects. The current study focused on acute short-term effects; hence, a follow-up study to assess long-term outcomes is strongly recommended. To build on the findings of this study, several types of research are necessary to address the translational challenges and validate the therapeutic potential of combining telmisartan and probiotics in CRC treatment. First, mechanistic studies should focus on elucidating the precise molecular pathways through which telmisartan and probiotics exert their anti-inflammatory, anti-angiogenic, and pro-apoptotic effects, especially in human-derived CRC cell lines and organoid models. Second, large-scale animal studies using models with microbiota and immune systems more analogous to humans, would provide a deeper understanding of the interaction between probiotics and host factors. Third, dose-optimization studies are needed to identify therapeutic concentrations that balance efficacy and safety for both telmisartan and probiotics. Finally, human clinical trials, beginning with phase I trials to evaluate safety and tolerability, followed by phase II and III trials to assess efficacy and long-term outcomes are crucial for confirming the clinical applicability of this combined treatment approach in diverse patient populations. These steps will ensure a comprehensive understanding and robust validation of the proposed therapy.

## Data Availability

All data are available from the corresponding authors upon request.

## Abbreviations

CRC: colorectal cancer
AT1R: Angiotensin-II Type 1 Receptor
CEA: Carcinoembryonic antigen
VEGF: Vascular endothelial growth factor
IL-6: Interleukin 6
TLRs: Toll-like receptors
COX-2: Cyclooxygenase-2
IL-12: Interleukin-12
NK: Natural killer
IFN-γ: Interferon-gamma
GM-CSF: Ggranulocyte-macrophage colony-stimulating factor
NF-κB: Nuclear factor-κB
DMH: 1,2-dimethylhydrazine
LB: Lactobacillus
TEL: Telmisartan
ELISA: Enzyme-linked immunosorbent assay
H&E: Hematoxylin and eosin
MMP-9: Matrix metalloproteinase-9
